# Single event response of ferroelectric spacer engineered SOI FinFET at 14 nm technology node

**DOI:** 10.1038/s41598-023-36952-1

**Published:** 2023-07-10

**Authors:** Baojun Liu, Jing Zhu

**Affiliations:** 1 Aviation Maintenance NCO Academy, Air Force Engineering University, Xinyang, 464000 Henan China; 2 Food College, Xinyang College of Agriculture and Forestry, XinYang, 464000 Henan China

**Keywords:** Electronic devices, Electrical and electronic engineering

## Abstract

The impact of spacer on the single event response of SOI FinFET at 14 nm technology node is investigated. Based on the device TCAD model, well-calibrated by the experimental data, it is found that the spacer presents the enhancement on single event transient (SET) compared with no spacer configuration. For single spacer configuration, due to enhanced gate control capability and fringing field, the increments in SET current peak and collected charge for HfO_2_ are the least with 2.21%, 0.97%, respectively. Four possible dual ferroelectric spacer configurations are proposed. The placement of ferroelectric spacer at S side and HfO_2_ spacer at D side brings to weaken SET with the variation in current peak and collected charge by 6.93%, 1.86%, respectively. The reason may be its enhanced gate controllability over the S/D extension region, which improves the driven current. With linear energy transfer increasing, SET current peak and collected charge present the trend of increase while the bipolar amplification coefficient reduces.

## Introduction

When an energetic particle strikes into the sensitive area of FinFET device, single event transient (SET) may be occurred by the diffusion and drift effects^1–3^. Due to the reduced parasitic capacitance from silicon-on- insulator (SOI) technology and the improved tolerance from thin fins, FinFET, with narrow silicon fin, combined with SOI and high *k*/metal gate stacked technology, brings benefits to radiation effects^2–4^. It was indicated that single event responses of FinFET may be significantly affected by ion hit angular, position and energy, supply voltage, device size and number of fins, technology node, and so on^3,5–7^. With the aggressive shrinking of device dimensions, spacer configuration and permittivity play the dominant roles in overall device performance^8–14^. It has been indicated that the device performance in terms of SS, current drivability, drain induced barrier lowering (DIBL) could be improved using an optimized spacer configuration^13,14^. However, at the best of our knowledge, no one has discussed the impact of spacer on single event response of FinFET.

In this paper, the single event response of spacer configuration and permittivity in SOI FinFET at 14 nm technology node is investigated. The rest of the paper is organized as follows: in Section II, FinFET device structure and fabrication processes flow are discussed. Section III presents the results and analyses of the impact of spacer on SET, followed by conclusions in Section IV.

## Finfet model

**Figure 1:**
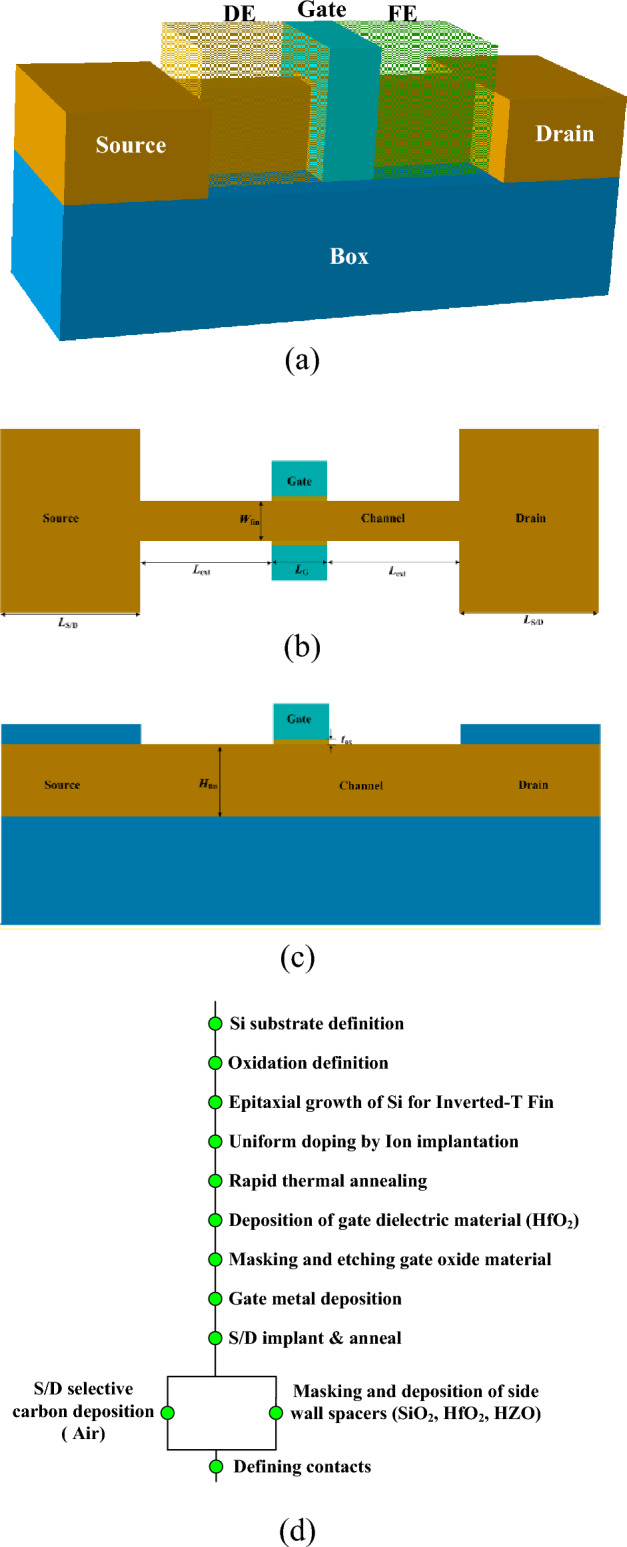
(**a**) FinFET structure with ferroelectric (FE) and dielectric (DE) spacer; (**b**) transverse structural section; (**c**) longitudinal structural section; (**d**) process flow for spacer configurations.

Based on TCAD, a 3D simulation model for n-type SOI FinFET at 14 nm technology node has been presented, shown in Fig. 1a–c. The gate is stacked with high-k dielectric material (HfO_2_) and metal contact (TiN). The length of the gate is 14 nm and the equivalent thickness of gate oxide is 0.5 nm. The width, height of fin is 10 nm, 18 nm, respectively. The length of S/D spacer is 33 nm. The channel silicon film is firstly uniformly p-type doped at a level of 5 × 10^15^ cm^−3^. The substrate silicon is uniformly p-type doped at a level of 1 × 10^15^ cm^−3^. Then, the S/D areas are Gaussian profile n-type doped with a peak value of 1 × 10^21^ cm^−3^. The extent S/D areas are also Gaussian profile n-type doped with a peak value of 8 × 10^19^ cm^−3^. The model fits well the experiment data^3^. In order to obtain accurate results, many physic models are included, such as, remote phonon scattering mobility model, Philips unified mobility model, Bohm quantum potential model, Shockley–Read–Hall (SRH) and Auger recombination models, heavy ion model, and so on^3^. For simulating the effects of high dielectric constants, polarization and hysteresis of ferroelectric materials (HZO), the Ferroelectric model is also set^15^. The permittivity of HZO is 22, and coercive field *E*_c_ = 1.0 MV/cm, remnant polarization *P*_r_ = 23 μC/cm^2^, saturated polarization *P*_s_ = 28 μC/cm^2^^16^. The fabrication processes flow of the spacer configuration is shown in Fig. 1d. ^17,18^

## Impact of spacer on set

The energetic particle hit the middle of fin between the drain and gate contact perpendicularly. The source and gate are connected to the ground and the drain of the device is set to 0.8 V. The linear energy transfer (LET) of the ion is 5 MeV cm^2^/mg. The strike has a radius of 10 nm and a delay time of 4 ps and the Gaussian profile has a characteristic time of 0.5 ps^3,19^. The run time is set to 1 ns.

### Single configuration spacer variation on SET

**Figure 2:**
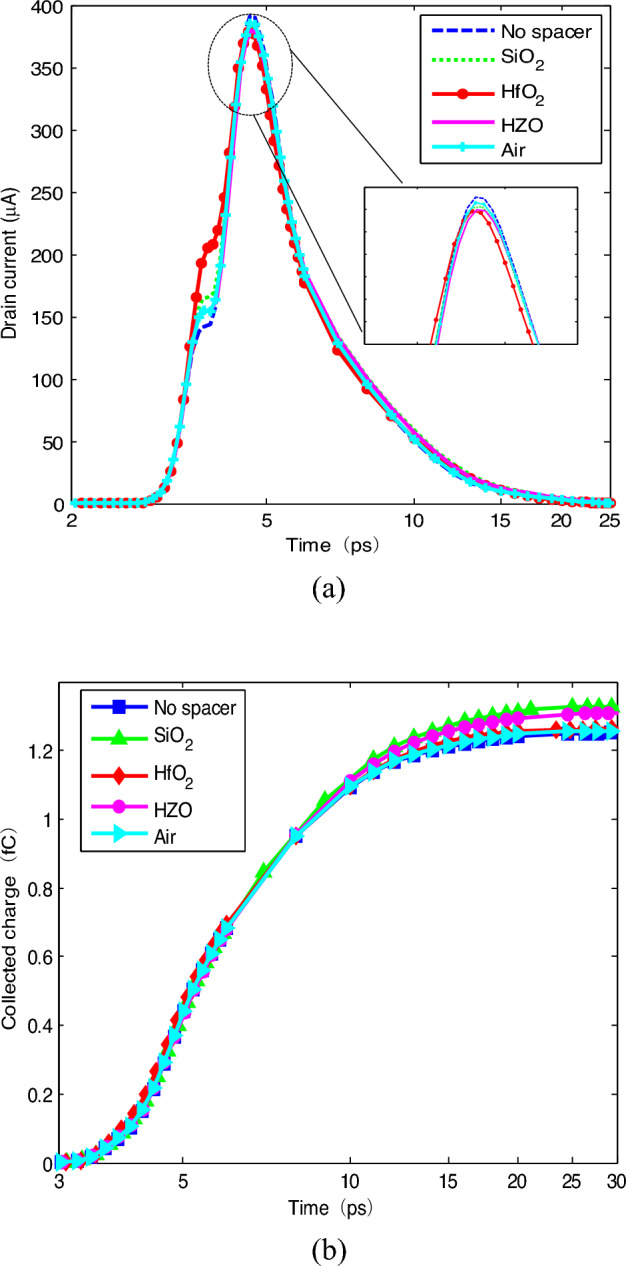
SET of single configuration spacers: (**a**) drain current; (**b**) collected charge.

In Ref.^8^, it has been indicated that FinFET with the high-*k* spacer material showed the concerning improvement of the performance parameters towards analog and RF design with a little compromise in speed of the device. The effects of the spacer materials with different dielectric constants on SET are investigated. For the single configuration spacer, low-*k*, high-*k* dielectric materials and ferroelectric materials are used, including Air (*k* = 1), SiO_2_ (*k* = 3.9), HfO_2_ (*k* = 25), HZO (*k* = 22). The direction of polarization is perpendicular to the fin surface by the same L-K parameters and thickness being used^13^. The drain currents and the collected charges are obtained, shown in Fig. 2. Here, the collected charge is achieved by integrating the simulated drain current over the transient duration.

It can be found that the spacer configuration impacts SET. As the spacer dielectric constant increases, the SET current peak presents in the trend of decrease. The relative decrement of SET current peak in single HfO_2_ spacer configuration is 2.21%. However, due to the spacer, the collected charges in all of the configurations are larger than that with no the spacer. The spacer exhibits non-monotonic trend of the collected charge. For low-*k* spacer, the collected charge increases with the dielectric constant increase. When the dielectric constant increases from 1 (Air) to 3.9 (SiO_2_), the relative increment in the collected charge varies from 0.15% to 6.43%. However, for high-*k* spacer, the trend in the collected charge presents different. The relative increments in the collected charge for HZO (*k* = 22) and HfO_2_(*k* = 25) spacer configurations are 4.92%, 0.97%, respectively. As well-known, the spacer generates the fringing field in the extension of source-to-gate and drain-to-gate. For the same physical thickness, the larger the dielectric constant of the material is, the smaller the equivalent thickness is. Therefore, it results in that the parasitic capacitances (*C*_GS_) formed between the source to the gate (or between the drain to the gate (*C*_GD_)) are larger and the generated surface potential of the channel is larger.

**Figure 3:**
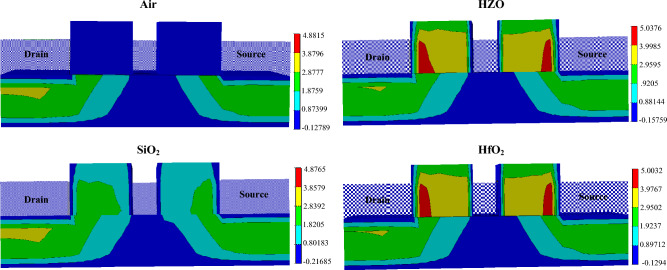
Electron quantum potential (in unit of V) after ion hit.

**Figure 4:**
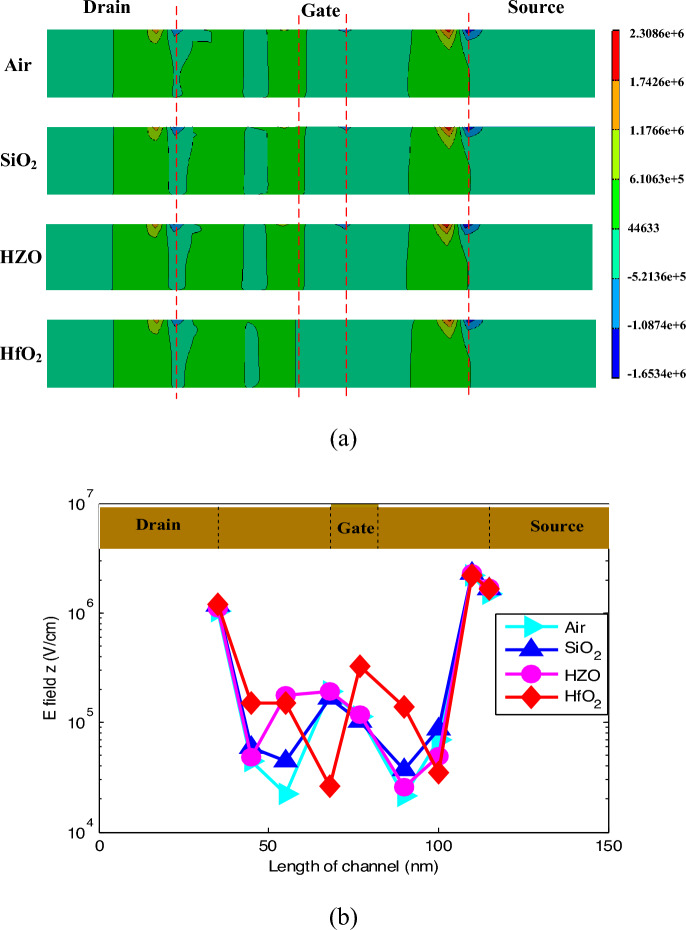
E field z (in unit of V/cm) after ion hit: (**a**) distribution profile; (**b**) maximum value along the channel.

Figure 3 shows the distribution of the electron quantum potential in the device, where the similar conclusion can be obtained. Also, due to the polarization effect of the ferroelectric material, the fringing field is stronger. The fringing field promotes the charge collection by the drain contact. On the other hand, the spacer increases the equivalent gate length. With the same physical thickness, the larger the dielectric constant of the material is, the greater the gate control capability of on the channel is. This leads to the stronger counteraction effect on the drift and diffusion of electron–hole pairs. Under the common effects of the fringing field and the gate controllability, the formed electric field distribution in the channel is shown in Fig. 4. It can be found that the electric field intensities in the devices of Air and HfO_2_ spacer configurations are relatively weaker, resulting in a smaller amount of charges collected by the drain and a weaker SET in the drain. Because of the strong electric field intensities in the devices of SiO_2_ and HZO, the drain will collect more charge. However, the fringing electric field is enhanced due to the ferroelectric material polarization, which increases the current driven capability^13^. It has been indicated that the device with a larger driven current was better immunity to SET^3,6^. This means that the increase of the driven current leads to the decrease of the collected charge by the drain.

### Dual configuration spacer variation on SET

**Figure 5:**
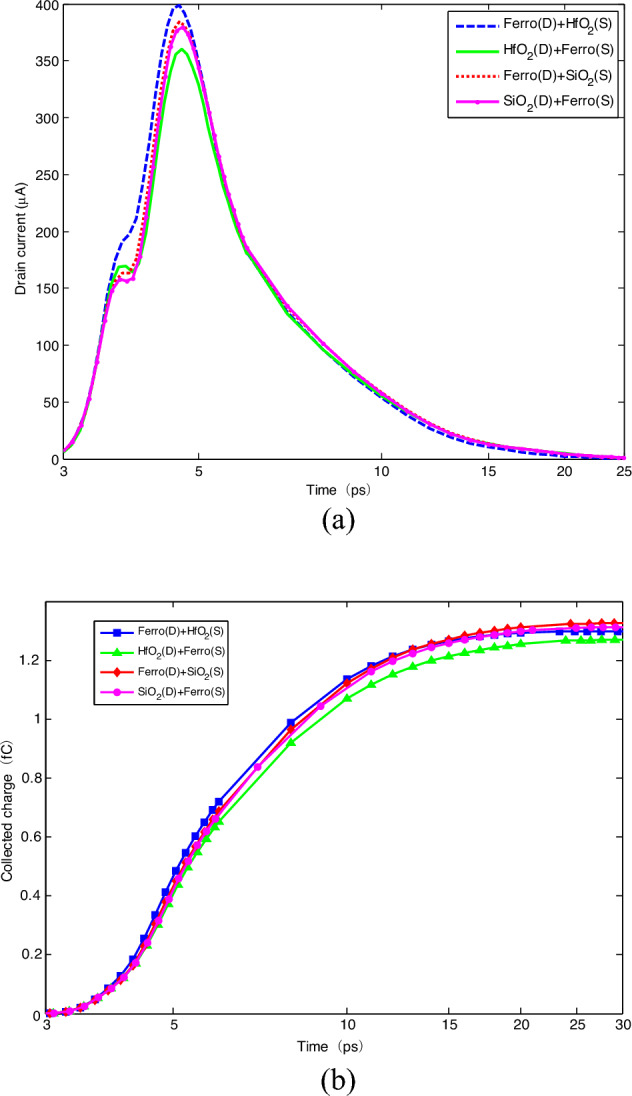
SET of dual configuration spacers: (**a**) drain current; (**b**) collected charge.

It has been indicated that ferroelectric spacer strongly couples the gate fringing fields between the gate and S/D extension, which impacts the performance of the device^13^. In order to improve the performance of the device with spacer to harden SET, four possible dual spacer materials configurations are proposed, based on the spacer alignment, i.e., (D1) D-side HfO_2_ and S-side HZO; (D2) D-side HZO and S-side HfO_2_; (D3) D-side SiO_2_ and S-side HZO; (D4) D-side HZO and S-side SiO_2_. The drain currents are achieved, shown in Fig. 5.

**Figure 6:**
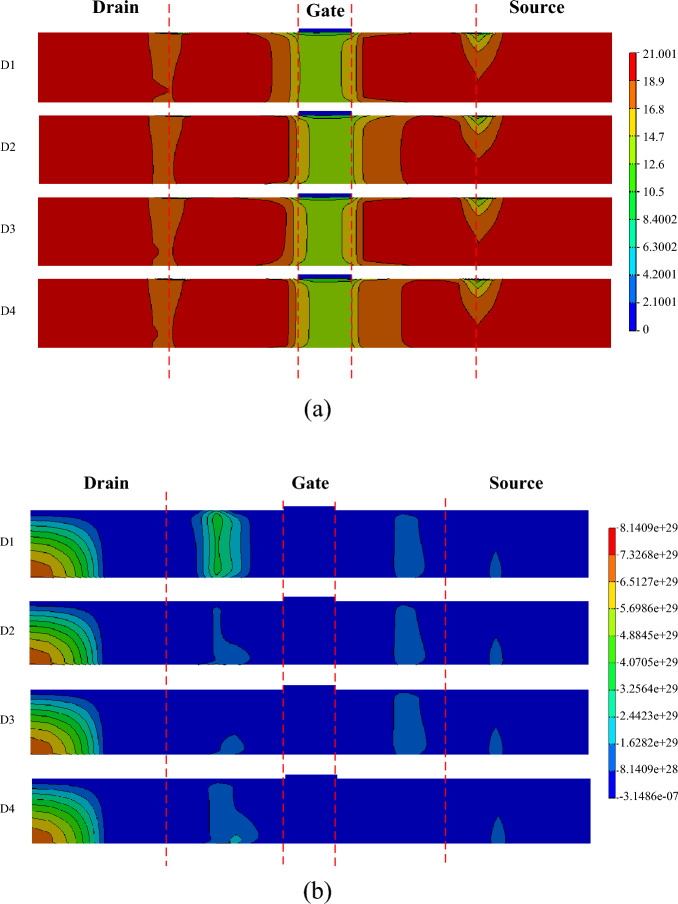
Variations of electron concentration and recombination rate along the channel after the ion hit: (**a**) electron concentration (/cm^3^); (**b**) recombination rate (/cm^3^s).

It is clear that the SET current peaks decrease for D1 and D3 configurations compared with the other two configurations. The collected charges for D1 and D2 configurations are less than the other two configurations. It can be found that the configurations with D-side ferroelectric material will strengthen SET. The reason may be that due to the polarization effect, D-side ferroelectric material promotes the ionization electron–hole pairs collected by the drain contact while S-side ferroelectric material attracts some electron–hole pairs to the source contact, leading to less collected charges. Figure 6 shows the recombination rate and the electron concentration along the channel after the ion hit the devices with different spacer configurations. It can be found that on the side with ferroelectric spacer, such as S-side at D1, D-side at D2, shown in Fig. 6a, the electron concertation is more than that on the side with HfO_2_ or SiO_2_. The fact is consistent with the above mentioned reason. Due to the larger recombination rate in D1 configuration, shown in Fig. 6b, the amount of the collected charge by the drain at D1 is less.

Compared with no spacer, the collected charge increases by relative 3.74%, 9.68% for D2 and D4 configurations while that increases by relative 1.86%, 5.12% for D1 and D3 configurations, respectively. As mentioned above, the larger the dielectric constant of the spacer is, the more the gate control capability is. It results in decreasing the current peak and the collected charge. Compared with no spacer, the relative variation of the current peak for D1 and D2 is 6.93%, 2.98%, respectively. However, the relative decrement of the current peak for D3 and D4 is 1.97% and 0.87%, respectively. Therefore, D1 configuration is the best one to harden SET.

**Figure 7:**
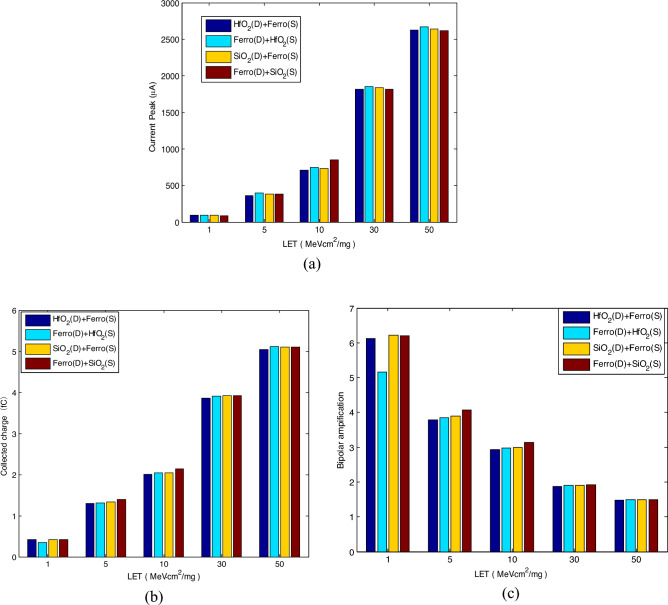
Influence of LET on SET: (**a**) current peak; (**b**) collected charge; (**c**) bipolar amplification coefficient.

Figure 7 shows the impact of LET on SET with different spacer configurations. The range of LET is set from 1 MeV cm^2^/mg to 50 MeV cm^2^/mg. Here, the bipolar amplification coefficient is achieved by the ratio between the collected charge and the deposited charge, which is obtained by considering the Gaussian distribution of the ion track and the 3D geometry of the silicon body^3^.

It is clear that as LET increases, the SET current peak and the collected charge increase while the bipolar amplification coefficient decreases. When LET increases from 1 MeV cm^2^/mg to 50 MeV cm^2^/mg, the increment of the current peak for four configurations is 2538.0μA, 2576.5μA, 2532.6μA, 2549.3μA, respectively. Also, the increment of the collected charge for four configurations is 4.62fC, 4.76fC, 4.68fC, 4.68fC, respectively. The decrement of the bipolar amplification coefficient for four configurations is 4.64, 3.66, 4.71, 4.73, respectively. There is no significant variation in bipolar amplification coefficient for both configurations found when LET is larger than 30 MeV cm^2^/mg. However, when LET is smaller than 30 MeV cm^2^/mg, the bipolar amplification coefficient for D1 and D2 is always less than the other configurations. It can be concluded that SET for D1 configuration is weaker than the other configurations. Compared with no spacer^3^, the bipolar amplification effect for spacer configurations is strengthened at LET lower than 10 MeV cm^2^/mg while the bipolar amplification effect is reduced at LET more than 10 MeV cm^2^/mg. This means that the spacer improves the collection of the charge. Also, it reduces the collected charge when LET is more than 10 MeV cm^2^/mg.

## Conclusion

With technology node scaled down, spacer configuration plays an important role in the overall device performance. Based on the calibrated 3D model for SOI FinFET at 14 nm technology node, the impact of spacer on SET is investigated for the first time. The single event responses in terms of current peak, collected charge and bipolar amplification coefficient for different spacer configurations and permittivity are obtained. The impacts of single spacer material and dual spacer placement with ferroelectric material on SET are analyzed and the potential mechanisms are also discussed. For single spacer configuration, SET for HfO_2_ spacer presents less increment in current peak and collected charge compared with no spacer configuration. For dual ferroelectric spacer configurations, the placement of FE spacer at the S side enhances the gate controllability over the extension region and thus improves the drive current, which weakens the single event response. The study of the single event response of all the possible ferroelectric spacer configurations in FinFET provides a great insight into the application of the device with enhanced the capability to harden SET under radiation environment.

## Data Availability

All data generated or analyzed during this study are included in this published article.
